# Multiple magnetoelectric coupling effect in BaTiO_3_/Sr_2_CoMoO_6_ heterostructures

**DOI:** 10.1038/s41598-017-03876-6

**Published:** 2017-06-20

**Authors:** Chang Liu, Wenhui Wan, Sai Gong, Hongbin Zhang, Wei Guo

**Affiliations:** 1 School of Physics, Beijing Institute of Technology, Beijing, 100081 China; 2 Kunming Institute of Physics, Kunming, 650223 China; 3 Department of Materials and Geosciences, TU Darmstadt, Darmstadt, 64287 Germany

## Abstract

Due to the demand of controlling magnetism by electric fields for future storage devices, materials with magnetoelectric coupling are of great interests. Based on first-principles calculations, we study the electronic and magnetic properties of a double perovskite Sr_2_CoMoO_6_ (SCMO) in a hybrid heterostructure combined with BaTiO_3_ (BTO) in different polarization states. The calculations show that by introducing ferroelectric state in BTO, SCMO transforms from an antiferromagnetic semiconductor to a half-metal. Specially, altering the polarization direction not only controls the interfacial magnetic moment, but also changes the orbital occupancy of the Co-3*d* state. This novel multiple magnetoelectric coupling opens possibilities for designing new type of spintronic and microelectronic devices with controllable degree of freedom of interfacial electrons in the heterostructures.

## Introduction

Multiferroics has attracted extensive research interests because magnetoelectric (ME) coupling effect enables control of magnetic properties via electric fields, and vice versa^1–3^. These superior characteristics possess wide range of possible novel applications, such as ME actuators, sensors, and high density nonvolatile memory devices^4–7^. Because of the relatively high ME operation temperatures and remarkable performance among the multiferroic family, composite multiferroic heterostructures, which are composed of magnetic materials and ferroelectric oxides, have been widely studied^8–11^. In general, three types of physical mechanism of ME coupling in such structures have been proposed^9, 12^, including strain mediated^13–17^, charge mediated^18–20^, and exchange interaction mediated mechanisms^21, 22^. Under such theoretical guidance, many efforts have been focused on searching new multiferroic systems with novel properties and high performance^11, 23–25^.

The orbital degree of freedom plays an important role in electronic structure and magnetic ordering and hence has a significant effect on physical properties^26, 27^. Usually, the orbital occupancy and shape can be modulated through introducing strain, but it is hardly controllable and inevitably introduces defects to materials. Very recently, Song *et al*. found in their experiment that the interfacial orbital occupancy can be partially changed under electric field in BaTiO_3_/La_2/3_Sr_1/3_MnO_3_ multiferroic heterostructures^28, 29^, providing an orbital mediated new ME coupling mechanism. However, theoretical study on this new type of ME coupling is rare, and new materials with better performance are vital for future applications.

In this paper, by means of first-principles calculations, we have studied three types of multiferroic heterostructures composed by ferroelectric material BaTiO_3_ (BTO) and antiferromagnetic (AFM) double perovskite Sr_2_CoMoO_6_ (SCMO) with different BTO polarization states. SCMO has a conventional double perovskite structure: the alternated CoO_6_ and MoO_6_ octahedrons with opposite rotations along [001] are arranged as a checkerboard configuration. Its antiferromagnetic order is along [101] direction and the Neel temperature is about 34 K^30, 31^. When introducing electric polarization in the BTO-SCMO heterostructure, SCMO transforms from AFM semiconductor to a ferromagnetic (FM) half metal. Via changing the direction of the dipole moments in BTO, a considerable variation of interfacial magnetization has been achieved, corresponding to a strong ME coupling. Particularly, we find that the orbital occupancy of interfacial Co^2+^ ions can also be controlled by the polarization. This new degree of freedom of interfacial electrons together with magnetism, conductivity and spin polarization, are all coupled with the electric polarization, leading to a multiple ME coupling effect. We believe that such coupling opens new perspectives in applications of composite multiferroic heterostructures for magnetic storage, microelectronic and spintronic devices.

## Results

**Figure 1:**
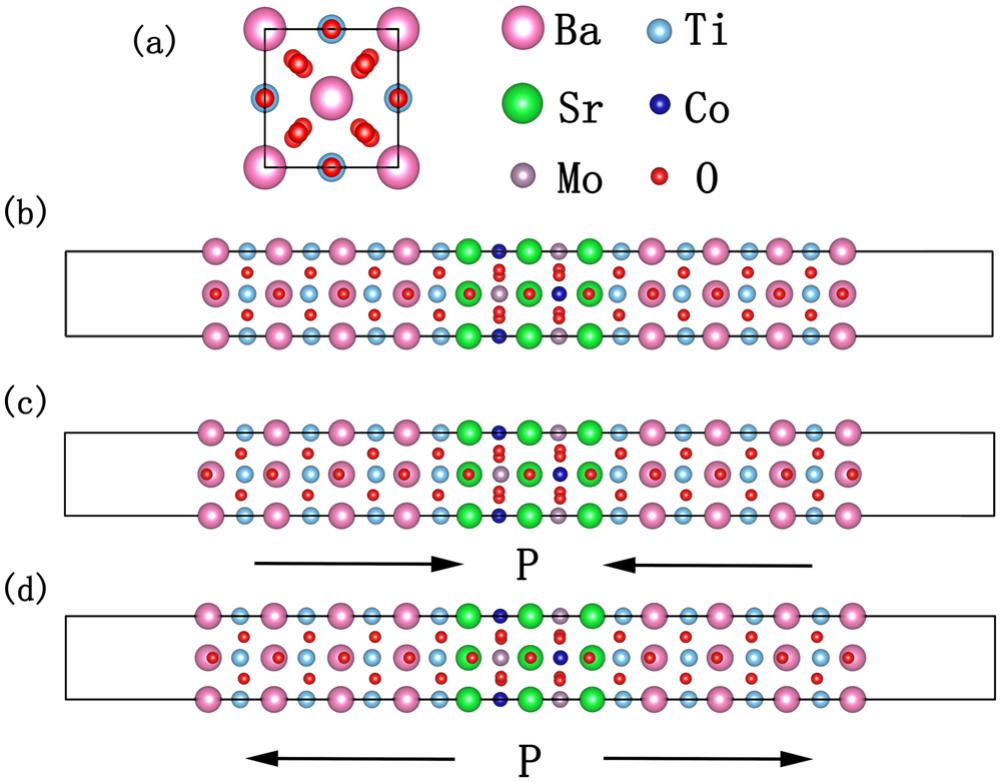
Atomic structure of three types of BTO-SCMO-BTO [001] multilayer configurations. Polarizations are induced by varying the Ti-O displacement in BTO. (**a**) Top view of the system. All three structures have been relaxed and the polarization orientation is marked by arrows. (**b**) Nonpolar system (NP), the BTO composition is pseudocubic with no polarization. (**c**) Type 1 polar system (P1), the polarization direction of BTO is towards SCMO. (**d**) Type 2 polar system (P2), the polarization direction is away from SCMO.

As shown in Fig. 1, three types of BTO-SCMO-BTO slab structures have been built along the *c*-direction with different BTO polarization states: Nonpolar system (NP) (Fig. 1 (b)); Type 1 polar system (P1) (Fig. 1 (c)) and Type 2 polar system (P2) (Fig. 1 (d)). More details for the heterostructures building can be found in the supplementary material (SM). We have calculated the total energy of ferromagnetic (FM) and antiferromagnetic (AFM) solutions in bulk SCMO and the configurations in Fig. 1. The energy difference between the two magnetic states ΔE_AFM−FM_ and the magnetic moments of Co and Mo atoms in the ground states are shown in Table 1. ΔE_AFM−FM_ is negative in bulk SCMO, indicating that AFM state is more stable than FM state. The magnetic moment M_Co _= 2.73 µ_B_ and M_Mo _= 0.04 µ_B_ for Co and Mo atoms, respectively, agreeing with previous experimental and theoretical studies^30–33^.

**Table 1:** Energy difference between ferromagnetic (FM) and antiferromagnetic (AFM) states in unit of meV per Co atom and the magnetic moments of Co and Mo atoms (M_Co_ and M_Mo_) in bulk SCMO and the NP, P1, P2 configurations.

	Bulk SCMO	NP	P1	P2
ΔE_AFM−FM_ (meV)	−9	−8	85	224
MCo(µ_B_)	±2.73	±2.73	2.69	3.10
MMo(µ_B_)	±0.04	±0.05	−0.42	0.01

For NP heterostructure in Fig. 1(b), the ΔE_AFM−FM_ and atomic magnetic moments on Co and Mo atoms are almost the same as in bulk SCMO, indicating that the SCMO slab is almost undisturbed and keeps the AFM state even in very thin film (~7.9 Å) and nonpolar BTO shows negligible effect on SCMO’s magnetic state. An interesting phenomenon arises when BTO generates ferroelectric instability in the P1 and P2 configurations, in which both of them turn into FM states from the bulk AFM solution. A considerable change in the atomic magnetic moment is observed (See Table 1), resulting in a large magnetoelectric coupling. For P1 configuration, the magnetic moment of Co is slightly decreased (from 2.73 µ_B_ to 2.69 µ_B_), while M_Mo_ varied from 0.05 µ_B_ to −0.42 µ_B_, corresponding to a large change of about 0.47 µ_B_. On the contrary, in P2, M_Co_ has a big change from 2.73 µ_B_ to 3.10 µ_B_, while M_Mo_ remains nearly zero as in NP system.

**Figure 2:**
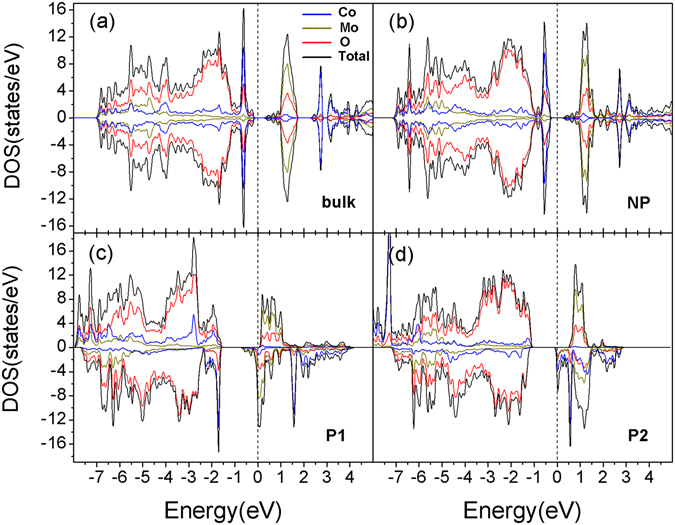
Local densities of states (DOS) of Co (blue line), Mo (dark yellow line), O (red line) atoms and Total DOS (black line) in (**a**) AFM bulk SCMO, (**b**) AFM NP, (**c**) FM P1 and (**d**) FM P2 configurations. Up- and down-spin DOS are shown in positive and negative values, respectively. The dotted lines correspond to Fermi level (*E*_*F*_).

To understand the nature of the polarization induced magnetism change, we have calculated the electronic structure and provide the densities of states (DOS) of bulk SCMO and heterostructures of BTO-SCMO-BTO in Fig. 2 (a) and Fig. 2 (b)–(d) in their ground states, respectively. BTO is a large gapped (about 3.3 eV) semiconductor, it has little contribution to the electronic states near *E*_*F*_, so we only show the local DOS of atoms in SCMO; the local DOS from Sr atom is not shown in Fig. 2 for the same reason. For bulk SCMO in the AFM state (Fig. 2(a)), we find that it is a semiconductor with a small gap of about 0.4 eV, the valence band maximum (VBM) is composed of Co-3*d* and O-2*p* electron states while the conduction band minimum (CBM) mainly contains Mo-4*d* and O-2*p* states. There is no big difference in the DOS between the NP configuration (Fig. 2 (b)) and bulk SCMO, except that the band gap becomes ~0.1 eV larger in NP system. This is probably due to the quantum confinement in ultrathin films^34^. When polarization is induced in the P1 (Fig. 2(c)) and P2 (Fig. 2(d)), interestingly, both systems show half-metallic character where one spin channel (majority-spin) behaves like an insulator with a finite gap, while the other spin channel (minority-spin) has non-zero DOS at *E*_*F*_. The magnetic moment variation in Mo and Co atom between different polar states can again be understood by comparing their DOS. As mentioned before, the CBM is mainly composed of Mo 4*d* electron states and system is semiconducting in NP. However, in P1, the minority spin of Mo CBM state is partially filled just below the *E*_*F*_, while the orbitals with majority spin are still unoccupied. Hence Mo produces a negative moment of −0.42 µ_B_. While in P2, the Mo CBM state is still unoccupied, therefore results in a nearly zero M_Mo_ like in NP.

For both P1 and P2 structures, the majority spin of Co is fully occupied while the minority spin only partially filled below the *E*_*F*_, such difference contributes to a net magnetic moment of about 3 µ_B_ where a small part of minority states gets occupied in P1, giving rise to the decrease in M_Co_ (See Table 1). However, the obvious increase of M_Co_ from P1 to P2 seems to be related to the different configuration of Co minority spin state in P2 system. There is a big difference in Co states between P1 and P2 especially in minority-spin channel. The peak of Co states at −1.7 eV in P1 shifts upward to 0.6 eV in P2. Meanwhile, the sharp peak around 1.5 eV in P1 system disappears in P2. This phenomenon may come from a variation of orbital occupancy in Co minority-spin state, and we will analysis this phenomenon next.

**Figure 3:**
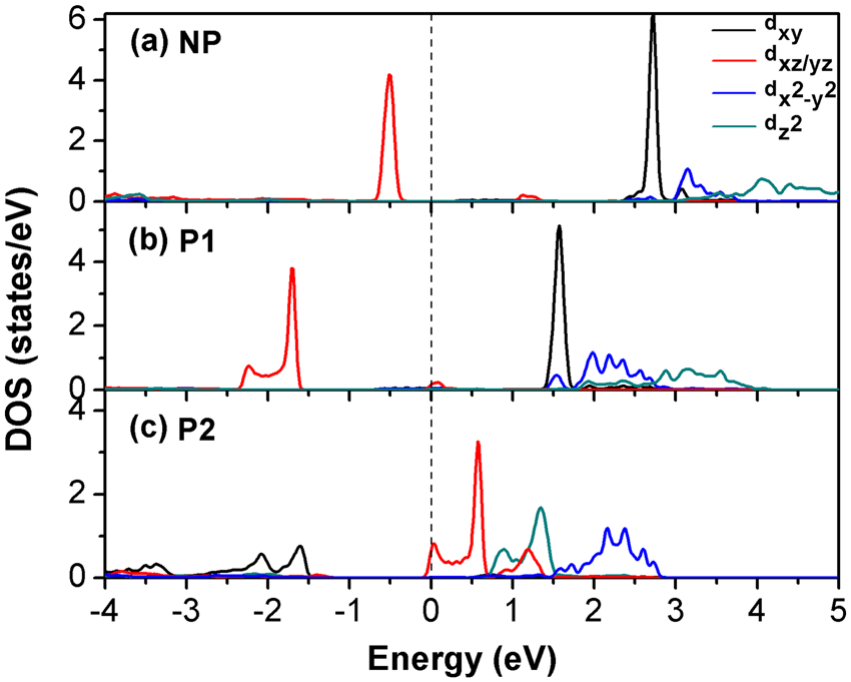
Projected Densities of states (PDOS) per atom of Co minority spin states in NP (**a**), P1 (**b**) and P2 (**c**). *d*_*xy*_, *d*_*xz*_/*d*_*yz*_, ${d}_{{x}^{2}-{y}^{2}}$, ${d}_{{z}^{2}}$ orbitals are shown as black line, red line, blue line and dark cyan line, respectively. The *d*_*xz*_ and *d*_*yz*_ orbital are degenerated due to the in-plane symmetry, so we only show one of them. The dotted lines correspond to Fermi level (*E*_*F*_).

We have calculated the projected density of state (PDOS) of the minority spin Co-3*d* electrons in NP (Fig. 3 (a)), P1 (Fig. 3 (b)) and P2 (Fig. 3 (c)). As Co ions are located in an octahedral coordination with the oxygen ions, the crystalline field causes an energy split between the *t*_*2g*_ and *e*_*g*_ orbitals. For both NP and P1, the *d*_*xz*_ and *d*_*yz*_ orbitals (the red curves in Fig. 3), which belong to *t*_*2g*_ symmetry, are almost completely filled while the other three minority spin orbitals, *d*_*yz*_, ${d}_{{z}^{2}}$ and ${d}_{{x}^{2}-{y}^{2}}$, are almost empty. This corresponds to an electron configuration of 3*d* ^7^ (*t*_*2g*_^*5*^ *e*_*g*_^2^) that two *t*_*2g*_ electrons occupy the minority spin state. The *E*_*F*_ in Fig. 3(b) crosses a small part of the Co CBM state and decreases the M_Co_, turning this system into half-metallic state. In P2 (Fig. 3c), however, the ordering of *d*_*xy*_ and *d*_*xz*_/*d*_*yz*_ orbitals is changed. The occupied states compose mostly of *d*_*xy*_ orbital, and the *d*_*xz*_/*d*_*yz*_ peak is pushed above *E*_*F*_. In this new electron configuration, less than 2 electrons are occupied in Co 3*d* minority spin state, while the majority-spin states are still fully filled, therefore the M_Co_ is increased in P2.

**Figure 4:**
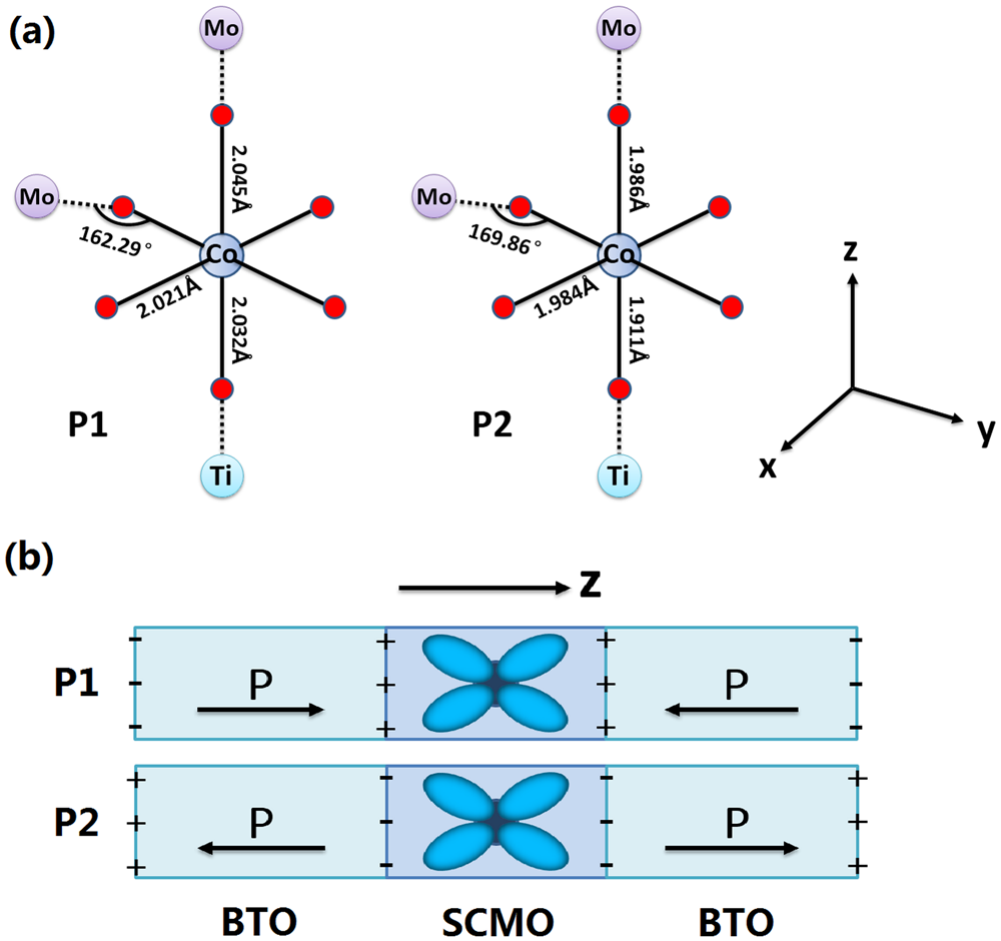
(**a**) Bond length and bond angle of Co-O octahedron in P1 and P2. The red small balls denote O ions and black lines denote Co-O bonds while the dotted lines are Mo-O and Ti-O bonds. (**b**) Schematic of different bound charges distribution in P1 and P2. Distribution of Co *d*_*xz*_ electron is shown in the SCMO region.

There are two possible origins for the variation of orbital occupancy in Co 3*d* electrons from P1 to P2. The first one is due to the CoO_6_ octahedral distortion and rotation. As shown in Fig. 4(a), The Co-O bond lengths in P1 are 2.021 Å (in-plane), 2.045 Å (Co-O-Mo out-of-plane) and 2.032 Å (Co-O-Ti out-of-plane), respectively. While in P2, all the Co-O bond lengths become shorter, leading to the in-plane bond length of 1.984 Å, and the out-of-plane values are 1.986 Å (Co-O-Mo) and 1.911 Å (Co-O-Ti), respectively. We note that in P2 the out-of-plane compression of Co-O octahedron is stronger than that of in-plane because of the displacement of the O ions under different ferroelectric polarizations. Such distortions will enhance the crystalline field splitting and raise the energy of *d*_*xz*_/*d*_*yz*_ orbitals. On the other hand, the in-plane Co-O-Mo bond angle gets larger in P2 than in P1. This octahedral rotation makes ${d}_{{x}^{2}-{y}^{2}}$ orbital closer to the in-plane O anions while *d*_*xy*_ is away from them. Therefore, the energy of ${d}_{{x}^{2}-{y}^{2}}$ orbital will rise up and the *d*_*xy*_ orbital energy will get lower at the same time. In brief, changes of the Co-O bond lengths raise the *d*_*xz*_/*d*_*yz*_ orbital energy while changes of the Co-O bond angle lower *d*_*xy*_ state energy, resulting in the different orbital occupancy in P2. The second reason for this effect is the influence of an electric field caused by different signs of bound charges at the interfaces^35^. In the P1 case, the electric dipole moment is pointing to the central SCMO (Fig. 4b), hence there are positive bound charges at the interfaces which attract electrons in *d*_*xz*_/*d*_*yz*_ states and reduce the orbital energy. On the contrary, in the P2 case, negative bound charges will raise the energy of *d*_*xz*_/*d*_*yz*_ states. The *d*_*xy*_ orbital does not get much influence in this mechanism because it is located in the *xy* plane, away from the bound charges. As a result, the change in the Co-O octahedron structure and different interface bound charges together change the orbital occupation in P2.

The polarization induced half-metallic property may be responsible for the magnetism transition between the AFM and FM states. When SCMO turns into the half-metallic state due to the polarization from BTO, hopping interactions between the localized electrons and the conduction electrons cause a large spin splitting of the effective Mo *d* band^36, 37^, resulting in a strong FM coupling between Co^2+^ ions via the induced spin-polarized mobile electrons. It has been proposed that the oxygen-deficient SCMO has a strong FM state (T_C_ = 350 K to 370 K) with a dramatic increase of conductivity^32^. This is also clear evidence that conducting electrons may induce FM magnetic coupling between Co^2+^ ions via the double-exchange mechanism in SCMO. Unfortunately, as the Neel temperature of SCMO is only about 34 K, it is therefore difficult to achieve the AFM−FM transition at room temperature. However, it is possible that double perovskites that have higher Neel temperature can be found through rational material design, and our approach provides a possible way to realize controllable AFM−FM transition though magnetoelectric coupling.

We have also tested a structure with thicker SCMO layers (1.5 u.c. SCMO + SrO) in P2 type of BTO polarization. We find that M_Co_ equals to 3.06 µ_B_ near the interface; while for the Co atom in the center of SCMO, M_Co_ is reduced to 2.96 µ_B_, indicating that the moment near the interface is mostly affected. Furthermore, we perform a calculation of a new BTO-SCMO polar state where both BTO polarizations point to the same direction (see Fig. S5 in Supplementary material). The magnetic response near the interface resembles the characters in P1 and P2 depending on whether the BTO polarization pointing to or away from SCMO (more details can be found in Supplementary material). By switching electric fields between SCMO and BTO, one can change the polarization state between P1, P2 and this new polar state and thereby control the system magnetization. The resulting surface ME coefficient α_s_ can be estimated by formula^38^ *μ*_0_Δ*M* = *α*_*s*_ *E*, where Δ*M* is the range of magnetization change, *E* is the strength of applied electric field and *μ*_0_ is the vacuum permeability. Taking Δ*M* = 0.84 *μ*_*B*_/*a* ^2^ (*a* is the lattice constant of SCMO) and assuming the coercive field of BTO *E*_*c*_ = 100 *kV*/*cm*, we obtain a ME coefficient *α*_*s*_ ≈ 3.1 × 10^−10^ *Gcm* ^2^/*V* which is comparable to that in Fe/PbTiO_3_ system as reported by Lee *et al*.^39^.

In summary, we have demonstrated multiple magnetoelectric effects in the BTO/SCMO/BTO heterostructures based on first-principles calculations. It is shown that the polarization state in BTO can be used to tailor the magnetic ground state, interfacial magnetic moments and conductivity of SCMO. More importantly, switching polarization direction changes the orbital occupancy of the Co-3*d* states drastically, which is originated from the distortion of the CoO_6_ octahedron in addition to the bound charges at the interface. Our results not only provide a novel ME system with tunable multiple magnetoelectric effects, but also present a broad opportunity to realize applications of orbital degree of freedom in spintronic and microelectronic devices.

## Methods

We have performed first-principles calculations of the electronic structures for three different BaTiO_3_/Sr_2_CoMoO_6_ [001] multilayer heterostructures (Fig. 1) using the Vienna Ab initio Simulation Package (VASP)^40, 41^ with the projector augmented wave (PAW) method generated pseudopotentials^42, 43^. For all types of multilayers, a vacuum layer of 15 Å were used with a BaO surface termination as shown in Fig. 1. BTO layers close to the vacuum are fixed to their bulk positions and are more than 1 nm away from the BTO-SCMO interface. We have tested that the effects from such artificial surface states are negligible. The in-plane lattice constants are fixed to the experimental value of the bulk SCMO (5.565 Å)^30^ which is slightly smaller than $\sqrt{2}$ times of BaTiO_3_’s lattice (3.991 Å in experiment)^44^, the induced compressive strain in BTO can therefore suppress the in-plane ferroelectricity instability^45^. Several optimizing processes have been made to eliminate possible artificial strains along c-direction. Details about the BTO-SCMO interface construction are shown in the supplementary material (SM). The convergence criterion was set to 10^−5^ eV for the self-consistent electronic minimization and 0.03 eV/Å for forces during the ionic relaxations. Since Co has 3*d* valence electrons, the on-site Coulomb interaction has been taken into account by using the LSDA+U approach^46^. We have used U = 5.0 eV and J = 0.89 eV for Co atom because they well reproduced the AFM semiconductor state in SCMO^33^. After careful convergence tests, we have chosen a cutoff energy of 530 eV for the plane wave basis and a k-mesh grid of 13 × 13 × 1 for Brillouin zone sampling. The tetrahedron method was used for the Brillouin zone integration. More calculation details can be found in the supplementary material (SM).

## Electronic supplementary material

Supplementary information.
