# Supplementary material for: Multiple magnetoelectric coupling effect in BaTiO3/Sr2CoMoO6 heterostructures
Source: Sci Rep. 2017 Jun 20;7:3856. doi: 10.1038/s41598-017-03876-6 (PMC5478603; doi:10.1038/s41598-017-03876-6)
Supplement: Supplementary file 1 — Supplementary information. [file 41598_2017_3876_MOESM1_ESM.pdf]

Multiple magnetoelectric coupling effect in $BaTiO_3/Sr_2CoMoO_6$ heterostructures

Chang Liu,[1,2] Wenhui Wan,[1] Sai Gong,[1] Hongbin Zhang,[3,*] Wei Guo[1,*]

[1]*School of Physics, Beijing Institute of Technology, Beijing, 100081, China*

[2]*Kunming Institute of Physics, Kunming, 650223, China*

[3]*Department of Materials and Geosciences, TU Darmstadt, Darmstadt, 64287, Germany*

** Corresponding author: weiguo7@bit.edu.cn, hzhang@tmm.tu-darmstadt.de*

The lattice constant of the (SCMO) is 5.565Å,[1] which is close to $\sqrt{2}$ times of $BaTiO_3$'s lattice (3.991Å).[1-3] Thus, the BTO-SCMO heterostructure has rather low lattice mismatch ~1%. To build the heterostructures, the in-plane lattice constants are fixed to the experimental value of the bulk SCMO (5.565Å), which is close to $\sqrt{2}$ times of commonly used perovskite substrates such as SrTiO3 (5.52 angstrom) and $SrRuO_3$ (5.56 angstrom) in experiment. While the out-of-plane lattice parameters are fully relaxed for bulk SCMO, cubic BTO (in paraelectric state) and tetragonal BTO (ferroelectric state), respectively. The relaxed structures were then used as the building blocks for the BTO/SCMO slabs. Under this constraint, we obtained a large polarization value of 0.55 C/m^2 for tetragonal BTO as calculated using the Berry phase approach,[4,5] in agreement with the fact that in-plane compressive strain can enhance the polarization along *c*-axis.[6,7]

Three types of BTO-SCMO-BTO slab structures have been built along the *c*-direction with different BTO polarity states: 1. Nonpolar system (NP) with the ideal cubic BTO structure to build the multilayer; 2. Type 1 polar system (P1), where the direction of the polarization in BTO points towards SCMO; 3. Type 2 polar system (P2), where the direction of polarization points away from SCMO, as

shown in the main paper. We have chosen the most stable interface (TiO_2-SrO configuration) and the surface is terminated in BaO layer. We first optimized the interlayer distance between BTO and SCMO in all three polarization states. After that, we fixed three outermost BTO monolayers to simulate the electrode's effect and optimized all the other layers to minimize the total energy of the whole system. We also set a comparison calculation with a $SrRuO_3$-BTO-SCMO slab (see Fig. S1) and optimized all the atomic coordinates to minimize the total energy and get the ground state structure, we find these two approach can obtain similar ferroelectricity at the BTO-SCMO interface. In consideration of the critical thickness of ferroelectric BTO, we attempted different thickness of BTO layers to build the polar slab. After structural relaxations, we found that a thickness of 4 unit cells is sufficient for maintaining the ferroelectric instability, which is consistent with previous calculations of the critical thickness in BTO.[3,8] So we finally constructed slabs as $(Ba_2Ti_2O_6)_4$-Sr_2O_2-Sr_2CoMoO_6-$(Ba_2Ti_2O_6)_4$. The experimental antiferromagnetic order for bulk SCMO is along [101] direction. However in the calculations, this type of antiferromagnetic needs to use a double sized SCMO supercell to build the heterostructure. In our calculations, we have used an antiferromagnetic order along [001] direction. We have compared the total energies between these two types of AFM order and find that although the [101] antiferromagnetic order is slightly more stable, the energy different is only 35meV/unit cell. It should not affect the conclusion of magnetic state transition between NP and P1/P2 structure. So we conclude that antiferromagnetic order along [001] is suitable in our calculations. Same model was also used in Etz et al.'s theoretical work[9].

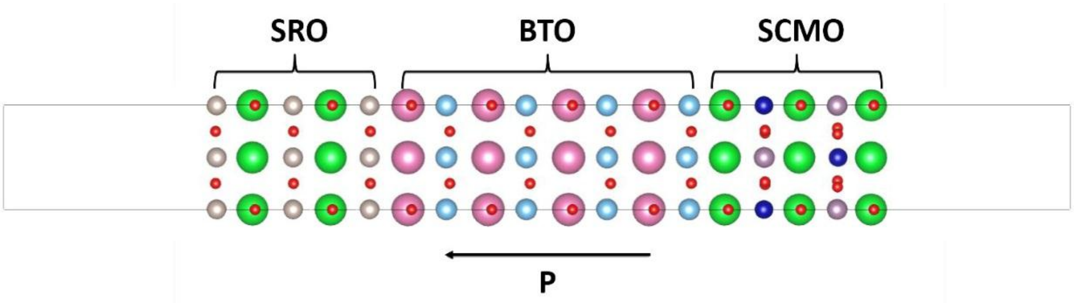

Supplementary Figure S1. Atomic structure of SrRuO3/BaTiO3/Sr2CoMoO6 trilayer, the polarization direction of BTO is shown by the arrow.

We also optimized bulk SCMO's structure and get a lattice constant of 5.524 Å, which is slightly smaller than the experimental value (5.565 angstrom). To test the influence of in-plane lattice constants, we recalculated our system with this lattice constant, and compare the result with the ones in our paper. We find that the magnetic moment of Co and Mo is not changed in NP structure. In P1 system, M_{Co} is changed from 2.69 μB to 2.68 μB while M_{Mo} from −0.42 μB to −0.43 μB. In P2 structure, M_{Co} changed from 3.10 μB to 3.09 μB and M_{Mo} remain unchanged. We also find that, the ordering switch between d_{xy} and d_{xz}/d_{yz} orbitals in P1 and P2 system remains the same (see Fig. S2). As a conclusion, the used experimental in-plane lattice constant has little influence to the basic physical conclusion.

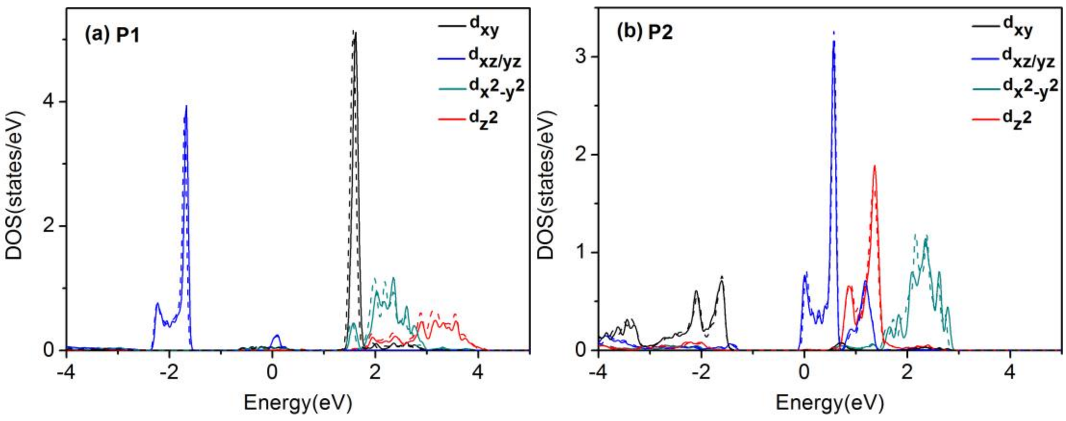

Supplementary Figure S2. Projected Densities of states (PDOS) per atom of Co minority spin states in P1 (a) and P2 (b), the in-plane lattice constant is fixed in the optimized bulk value of SCMO. d_{xy}, d_{xz}/d_{yz}, $d_{x^2-y^2}$, d_{z^2} orbitals are shown as black line, blue line, dark cyan line, and red line, respectively. The dotted line denotes Co PDOS (Figure.3 in the main manuscript) in experimental lattice constant.

In our model vacuum layers were used to separate BTO in periodic supercell so that we can alter polarization in each side of BTO independently without introducing extra electrode layers. To demonstrate that this model also gives reliable results, we build a bulk BTO/SCMO superlattice as shown in Fig. S3. There's no surface in this structure so that the influence of surface state can be excluded. We calculate the magnetic moment of Co and Mo atoms after structure relaxation. We find that at the left interface (corresponding to the interface in P1 structure), M_{Co} has the same value (2.69 μ_B) as in P1 structure, and M_{Mo} is −0.31 μ_B, a little smaller than in P1 (−0.42 μ_B). While in the right interface (corresponding to the interface in P2 structure), M_{Co} (3.04 μ_B) and M_{Mo} (−0.06 μ_B) has little change with their value in P2 (3.10 μ_B and 0.01 μ_B, respectively). Moreover, the orbital occupancy of Co atom is also changed in the right interface as the same as in the P2 system in the manuscript, as shown in the Co-PDOS figure below, the dotted line and solid line denote Co PDOS in polar BTO/SCMO heterostructures with and without surface state, respectively. The layers close to the vacuum are fixed to their bulk positions and are more than 1 nm away from the BTO-SCMO interface. Thus, the surface state has little effect on our conclusion, and the model in the manuscript is suitable for our research.

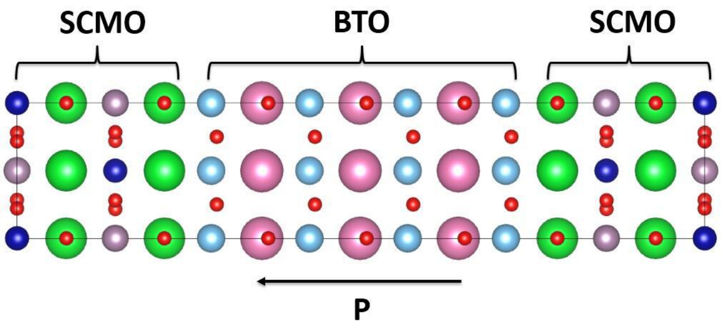

Supplementary Figure S3. BTO/SCMO supperlattice structure. There is no surface in this structure, the polarization direction of BTO are shown by arrow.

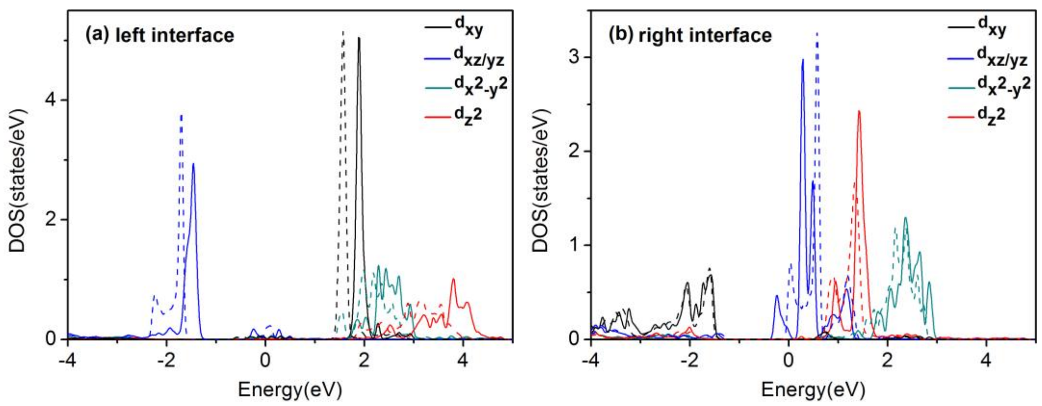

Supplementary Figure S4. Projected Densities of states (PDOS) per atom of Co minority spin states in left interface-P1 like (a) and right interface-P2 like (b) in the BTO-SCMO supperlattice shown in Fig. S3. d_{xy}, d_{xz}/d_{yz}, $d_{x^2-y^2}$, d_{z^2} orbitals are shown as black line, blue line, dark cyan line, and red line, respectively. The dotted line denotes Co PDOS (Figure.3 in the main manuscript) in P1 (a) and P2 (b) structures.

We also considered the structure where both sides of BTO polarizations point to the same direction, which is shown in Fig. S5. Actually, such polarization arrangement is included by our P1 and P2

models. In this situation, we find that at the left interface (where the polarization pointing towards SCMO), the magnetic moment of Co and Mo are 2.70 μ_B and −0.26 μ_B, respectively. This value is close to the moment in P1 structure. At the right interface (where the polarization pointing away from SCMO), the magnetic moment are 2.98 μ_B and −0.01 μ_B for Co and Mo atom, respectively. This value is also close to the P2 structure where M_{Co}=3.10 μ_B and M_{Mo}=0.01 μ_B. We also analyzed the occupancy of Co 3d state, and find that the d_{xy} and d_{xz}/d_{yz} orbitals switch in order between the left interface (resembles the P1 structure) and the right interface (resembles the P2 structure). As shown in Fig. S6, the solid line denotes Co PDOS in this new polar structure and the dotted line denotes the PDOS in P1 (a) and P2 (b) system. Therefore, the key factor of controlling the magnetism and orbital occupancy in SCMO is the polarization pointing direction near the interface. By adding electrodes at SCMO and BTO, we can change this polarization direction by applying electric field, thereby change the magnetism and orbital properties in SCMO.

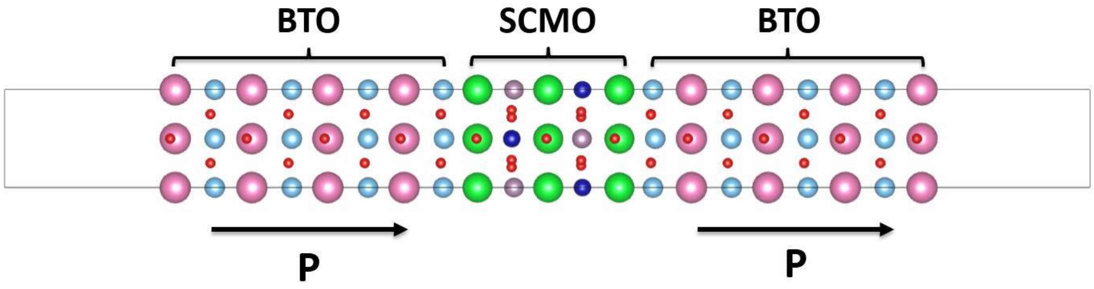

Supplementary Figure S5. Atomic structure of BTO-SCMO-BTO trilayer, where both side BTO polarizations point in the same direction, the polarization direction of BTO are shown by arrow.

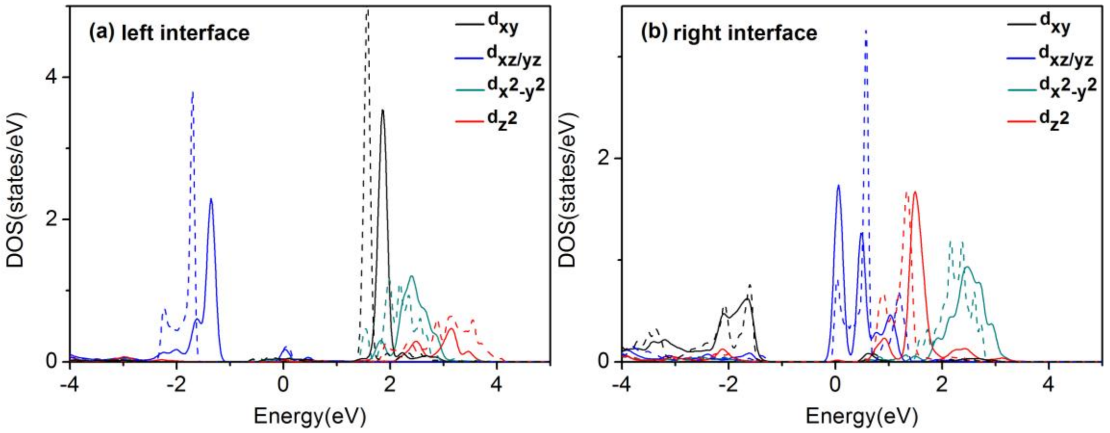

Supplementary Figure S6. Projected Densities of states (PDOS) per atom of Co minority spin states in left interface-P1 like (a) and right interface-P2 like (b) in the BTO-SCMO-BTO trilayers shown in Fig. S5. d_{xy}, $d_{xz}/d_{yz}, d_{x^2-y^2}$, d_{z^2} orbitals are shown as black line, blue line, dark cyan line, and red line, respectively. The dotted line denotes Co PDOS (Figure.3 in the main manuscript) in P1 (a) and P2 (b) structures.

1 Itoh, M., Ohta, I. & Inaguma, Y. Valency pair and properties of 1:1 ordered perovskite-type compounds Sr2MMoO6 (M = Mn,Fe,Co). *Materials Science and Engineering: B* **41**, 55-58, doi:http://dx.doi.org/10.1016/S0921-5107(96)01623-6 (1996).

2 Ivanov, S. A., Eriksson, S. G., Tellgren, R., Rundlöf, H. & Tseggai, M. The magnetoelectric perovskite Sr2CoMoO6: An insight from neutron powder diffraction. *Mater. Res. Bull.* **40**, 840-849, doi:10.1016/j.materresbull.2005.01.012 (2005).

3 Sai, N., Kolpak, A. M. & Rappe, A. M. Ferroelectricity in ultrathin perovskite films. *Phys. Rev. B* **72**, 020101 (2005).

4 Resta, R. Theory of the electric polarization in crystals. *Ferroelectrics* **136**, 51-55, doi:10.1080/00150199208016065 (1992).

5 King-Smith, R. D. & Vanderbilt, D. Theory of polarization of crystalline solids. *Phys. Rev. B* **47**, 1651-1654 (1993).

6 Pertsev, N. A., Zembilgotov, A. G. & Tagantsev, A. K. Effect of Mechanical Boundary Conditions on Phase Diagrams of Epitaxial Ferroelectric Thin Films. *Phys. Rev. Lett.* **80**, 1988-1991 (1998).

7 Diéguez, O., Rabe, K. M. & Vanderbilt, D. First-principles study of epitaxial strain in perovskites. *Phys. Rev. B* **72**, 144101 (2005).

8 Gerra, G., Tagantsev, A. K., Setter, N. & Parlinski, K. Ionic polarizability of conductive metal oxides and critical thickness for ferroelectricity in BaTiO3. *Phys. Rev. Lett.* **96**, 107603,

doi:10.1103/PhysRevLett.96.107603 (2006).

9 Etz, C. & Stoeffler, D. First principle study of the magnetism of Sr2CoMoO6-δ (δ = 0, 1/2) double perovskites. *The European Physical Journal B* **54**, 429-434, doi:10.1140/epjb/e2007-00017-7 (2007).
